# Southernmost Asia Is the Source of Japanese Encephalitis Virus (Genotype 1) Diversity from which the Viruses Disperse and Evolve throughout Asia

**DOI:** 10.1371/journal.pntd.0002459

**Published:** 2013-09-19

**Authors:** Xiaoyan Gao, Hong Liu, Huanyu Wang, Shihong Fu, Zhenyang Guo, Guodong Liang

**Affiliations:** 1 State Key Laboratory for Infectious Disease Prevention and Control, Collaborative Innovation Center for Diagnosis and Treatment of Infectious Diseases, National Institute for Viral Disease Control and Prevention, Chinese Center for Disease Control and Prevention, Beijing, People's Republic of China; 2 School of Life Sciences, Shandong University of Technology, Zibo, Shandong, People's Republic of China; University of Texas Medical Branch, United States of America

## Abstract

**Background:**

Although a previous study predicted that Japanese encephalitis virus (JEV) originated in the Malaysia/Indonesia region, the virus is known to circulate mainly on the Asian continent. However, there are no reported systematic studies that adequately define how JEV then dispersed throughout Asia.

**Methodology/Principal Findings:**

In order to understand the mode of JEV dispersal throughout the entire Asian continent and the factors that determine the dispersal characteristics of JEV, a phylogenetic analysis using Bayesian Markov chain Monte Carlo simulations was conducted on all available JEV E gene sequences in GenBank, plus strains recently isolated in China. Here we demonstrate for the first time that JEV lineages can be divided into four endemic cycles, comprising southern Asia, eastern coastal Asia, western Asia, and central Asia. The isolation places of the viruses in each endemic cycle were geographically independent regardless of years, vectors, and hosts of isolation. Following further analysis, we propose that the southernmost region (Thailand, Vietnam, and Yunnan Province, China) was the source of JEV transmission to the Asian continent following its emergence. Three independent transmission routes from the south to north appear to define subsequent dispersal of JEV. Analysis of JEV population dynamics further supports these concepts.

**Conclusions/Significance:**

These results and their interpretation provide new insights into our understanding of JEV evolution and dispersal and highlight its potential for introduction into non-endemic areas.

## Author Summary

Japanese encephalitis virus (JEV) probably originated in the Malaysia/Indonesia region. Currently, there are no systematic studies that adequately define how it subsequently dispersed throughout Asia. In this study, we demonstrate that JEV lineages can be divided into four endemic cycles, comprising southern Asia, eastern coastal Asia, western Asia, and central Asia. In each endemic cycle the source of virus was geographically independent regardless of year, vector, and host of isolation. The southernmost region (Thailand, Vietnam, and Yunnan Province, China) was identified as the most likely source of JEV transmission from its origin to the Asian continent. Based on the evidence, we identified three probable JEV dispersal routes from south to north. Analysis of JEV population dynamics further supports this view. Our results provide new insights into the understanding of JEV evolution and dispersal and highlight its potential for introduction into non-endemic areas.

## Introduction

Japanese encephalitis (JE) is arguably one of the most serious viral encephalitic diseases worldwide [1], [2]. According to the latest report of the World Health Organization, JE is endemic in 24 Asian and Oceanian countries, with an estimated 67,900 JE cases annually (the total morbidity rate is 1.8/100,000 population). An estimated 3 billion people live in countries where JE is endemic. Additionally, with increased international travel to JE endemic areas, more people are at risk of JE infection. Therefore, JE is not only an endemic disease in Asian and Oceanian countries, it could also potentially cause significant public health issues in non-endemic countries or regions and has the realistic possibility of becoming a serious global public health problem [1]–[4].

JEV is the prototype member of the JEV serogroup within the genus *Flavivirus*, family *Flaviviridae*. The viral genome is a positive-sense, single-stranded RNA that is approximately 11 kb in size. The genome carries a single open reading frame (ORF) encoding a polyprotein that is processed into three structural proteins [capsid (C), membrane (M), and envelope (E)] and seven nonstructural proteins (NS1, NS2A, NS2B, NS3, NS4A, NS4B, and NS5) [5], [6]. Phylogenetic analysis of JEV has shown that based on the E gene or the complete genome, JEV can be divided into five genotypes (G1–G5) [7], [8], [9].

JEV is maintained in nature in a cycle involving vertebrate hosts (including pigs and waterbirds) and *Culex* mosquitoes. *Culex tritaeniorhynchus* is the primary vector [10]. Pigs are important reservoir hosts of JEV. Migrating birds are thought to be an important factor in the dispersion of JEV to new geographical areas [11]. During the virus transmission cycle, mosquitoes become infected with JEV when they feed on infected pigs and birds. They replicate the virus and subsequently feed again, in some cases transmitting the virus to humans or horses which are incidental hosts of JEV.

JEV was predicted to have originated from the tropical Indonesia/Malaysia region because there is evidence that this region had all genotypes of JEV circulating, whereas only the more recent genotypes circulate in other areas [12]. However, the virus is currently known to circulate throughout Asia. These observations raise several questions. Firstly, what is the pattern and direction of JEV dispersal from the Indonesia/Malaysia region to the entire Asian continent? Secondly, what are the primary factors that determine the dispersal characteristics of JEV? Thirdly, what is the contribution to virus dispersal of migratory birds, seasonal winds, mosquitoes and other factors, such as temperature and rainfall? Resolution of these intriguing questions will not only inform science but will also provide guidance for public health authorities in the development of prevention and control strategies for JE. Previous reports on transmission patterns showed that JEVs in East Asia were introduced from South East Asia [13], and JEVs circulating in Japan were introduced from South East Asia and continental East Asia [14]. However, these studies were limited to local areas. Therefore, in the present study, together with the new JEV strains isolated in China, we analyzed all available sequences of the currently predominant genotype (G1) of JEV isolates that are widely dispersed over the Asian continent.

## Materials and Methods

### Virus cultures, RNA extraction, reverse transcription, polymerase chain reaction (PCR) amplification, and sequencing of new JEV strains from China

The E protein gene of 22 JEV strains newly isolated in China from 2005 to 2010 was sequenced. Among these 22 strains, two were isolated from Yunnan Province in 2005 and 2006; four were isolated from Guangxi Province in 2006, Henan Province in 2006, Shanxi Province in 2006, and Jiangxi Province in 2009, respectively; two were isolated from Liaoning Province in 2006 and 2007; three were isolated from Shandong Province in 2008 and 2009; three were isolated from Chongqing Municipality in 2008 and 2009; and eight were isolated from Hubei Province during 2008–2010. The isolation protocols have been described elsewhere [8]. Briefly, the viruses were amplified once by infecting *Aedes albopictus* C6/36 mosquito cells. After development of cytopathic effects (CPE), culture supernatants were harvested, and viral RNA was extracted using the QIAamp Viral RNA Mini Kit (Qiagen, Hilden, Germany). The purified RNA was used as the template for cDNA synthesis using Ready-to-Go You-Prime First-Strand beads (Amersham Biosciences, Piscataway, NJ, USA). The complete E gene was amplified using the following primers: JEV-Ef [5′-TGYTGGTCGCTCCGGCTTA-3′ (955–973)] and JEV-Er [5′-AAGATGCCACTTCCACAYCTC-3′ (2516–2536)]. Amplified products were examined by agarose gel electrophoresis (1%), purified using a QIAquick Gel Extraction Kit (Qiagen) and then sequenced directly.

The envelope sequences of these 22 newly isolated JEVs determined in the present study were deposited in GenBank under Accession Numbers JQ937333 and JQ937336–JQ937356 (Table S1).

### Construction of the data set for phylogenetic analysis

The most recent study showed that in evolutionary terms, the G1 JEV genotype is the youngest of five genotypes and is the dominant genotype circulating in Asia [15]. For studying the dispersing patterns of G1 JEV, totally 656 E sequences of JEV, with information regarding the isolation time and place, were downloaded from GenBank (as of May 1, 2012). ClustalX version 2.0.9 [16] was used to generate sequence alignments of 678 E gene sequences (including 22 newly contributed sequences). The dataset was screened for recombination using RDP3 (Recombination Detection Program3) and GARD (genetic algorithm for recombination detection) [17], [18]. No recombination events were identified (data not shown). Subsequently, in order to differentiate G1 from the other four genotypes, the Neighbor-joining method in Mega Version 5.05 [19] was applied for phylogenetic analysis. Finally, 359 E sequences of the G1 JEV genotype were obtained and comprised a sequence database for phylogenetic analysis (Table S1).

### Phylogenetic and phylogeographic analysis of genotype 1 JEV

The sequence database constructed above was analyzed using Bayesian Markov chain Monte Carlo (MCMC) method. The GTR+I+G was selected as the optimal nucleotide substitution model by MrModelTest [20]. The nucleotide substitution rate and divergence time of the most recent common ancestor (TMRCA) were estimated using the relaxed (uncorrelated lognormal) molecular clock model under a coalescent model of constant population size in the BEAST software package [21]. Demographic histories were inferred by Bayesian skyline reconstruction. The analysis was run through 600,000,000 generations to ensure sufficient mixing. Convergence of parameters was checked using TRACER and was indicated as effective sample size (ESS>200), and the maximum clade credibility (MCC) tree was built using TreeAnnotator with 10% burn-in (http://beast.bio.ed.ac.uk/). Statistical uncertainty was expressed for nodal support by 95% confidence intervals of the highest posterior density (HPD).

In order to infer the history of geographical dispersion of JEV, the Bayesian stochastic search variable selection (BSSVS) was used to provide evidence for statistically supported diffusion between state variables under BEAST v1.7.5 [22]. This method estimates the most probable state at each node in the MCC trees, allowing us to reconstruct ancestral positions for ancestral viral lineages along the tree. For phylogeographic reconstructions, each region was coded as a discrete trait. BSSVS output and surfaces representing uncertainty for continuous diffusion processes were formatted as KML using the SPREAD utility [23]. Determination of each locality was coordinated and performed using Google Earth v.6.2.2. Mapinfo was finally used to display the dispersal pattern of JEV based on the phylogeographic analysis.

## Results

### Spatial and temporal distribution of genotype 1 JEV

Based on the isolation sources of 359 G1 JEV strains, by 2010, the distribution of the G1 JEV genotype had shifted northward to a latitude of 45° (Japan) and westward to a longitude of 75° (India) and had covered almost all JEV endemic areas, including Australia, Thailand, Vietnam, Cambodia, India, Japan, Korea, Taiwan, and most regions of China (15 provinces). The Malaysian G1 JEV sequence was not included in the present study because only the PrM sequence was available. The 22 newly isolated JEVs contributed to knowledge regarding the geographical distribution of the G1 genotype in central Asia, a highly endemic area for JEV and added to strains isolated in China after 2005, but especially after 2009. A time span of 44 years was found for the isolation time of the 359 strains studied, from 1967, when the first G1 JEV genotype was isolated, to 2010. Additionally, the viral strains used were from various sources, including insect vectors (various mosquito species and midges) and host animals (pigs and human patients).

### Geographical characteristics of genotype 1 JEV based on phylogenetic analysis

**Figure 1:**
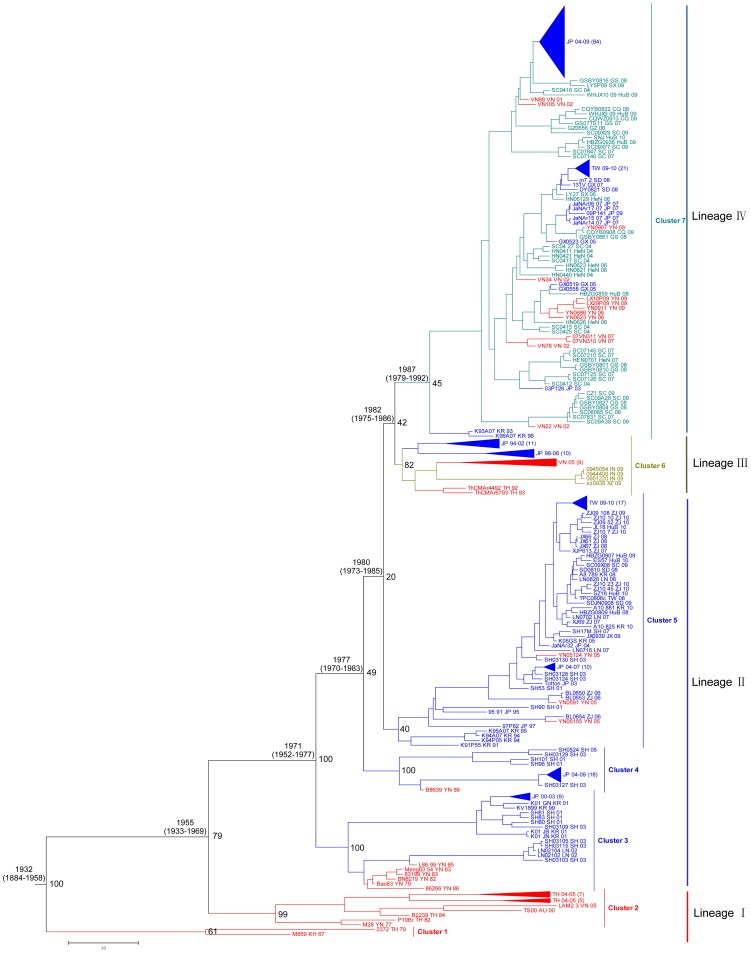
Maximum clade credibility tree for E gene sequences of genotype 1 JEV. Seven clusters were identified and estimated TMRCAs of these clusters (with their 95% HPD values in parentheses) are shown. Four endemic cycles were grouped based on the geographical locations of JEV strains in each cluster. Red, blue, olive and turquoise were used to mark strains from southernmost Asia, eastern coastal Asia, western Asia, and central Asia, respectively. Posterior probability values of each cluster and endemic cycles were showed in the right of the nodes. In order to present all available sequences in the tree, black triangles were used to condense strains with the same isolation place and similar isolation times.

The maximum clade credibility (MCC) tree of the 359 E sequences of JEV is shown in Figure 1. The tree showed that the G1 JEV genotype isolates can be divided into seven clusters according to their geographic isolation sites (designated clusters 1–7). They were further grouped into four lineages, lineage I (clusters 1 and 2), II (clusters 3–5), III (cluster 6) and IV (cluster 7). According to the geographic locations of the principal JEV strains in each lineage, four lineages were also designated as the southern Asia endemic cycle, the eastern coastal Asia endemic cycle, the western Asia endemic cycle, and the central Asia endemic cycle, respectively. Virus strains in the southern Asia endemic cycle were mostly derived from Vietnam, Thailand, Cambodia, Australia, and Yunnan Province in China. Strains in the eastern coastal Asia endemic cycle were mainly derived from Shanghai, Zhejiang, Liaoning, Shandong, Taiwan, Japan, and Korea; strains from southernmost Yunnan Province and those isolated after 2008 in Chongqing, Hubei, and Jiangxi of central China (such as JX0939, SZ18, JL18, ES57, HBZG0907, and HBZG0809) are also included in this endemic cycle. Most of the strains in the western Asia endemic cycle were obtained from Tibet and India in western Asia, and other strains were isolated in southernmost regions such as Thailand and Vietnam or Japan. The dominant strains in the central Asia endemic cycle were from the inland provinces in China, including Guizhou, Sichuan, Chongqing, Henan, Hubei, Gansu, and Shanxi. This latter cycle also contains a few strains from the southernmost part of Asia such as Vietnam and Yunnan Province in China, and the eastern coastal regions of Asia such as Shandong, Taiwan, and Guangxi in China, Japan, and South Korea.

### The source of genotype 1 JEV in the southernmost region of Asia based on phylogenetic analysis

The most recent common ancestor (TMRCA) for the G1 JEV genotype is estimated to have diverged approximately 78 years ago based on the Bayesian MCMC approach using E gene sequences. The resultant G1 JEV genotype first appeared in southernmost Asian regions, such as Thailand, Vietnam, and Yunnan Province in China and established endemic cycles in those regions.

Detailed analysis of the geographical locations of strains in the four endemic cycles showed that at least one strain of JEV isolated from southernmost regions of Asia, including Thailand, Vietnam, and Yunnan Province in China was present in eastern coastal Asia, western Asia, and central Asia endemic cycles (Figure 1, Table 1). For example, isolates from Yunnan Province in China were included in the eastern coastal Asia endemic cycle, isolates from Thailand and Vietnam were included in the western Asia endemic cycle, and isolates from Vietnam and Yunnan Province in China were included in the central Asia endemic cycle. Moreover, based on the chronological order of evolution, the strains isolated from the southernmost regions mostly occurred earlier than others and rooted those in each endemic cycle. However, those strains from other regions of Asia were found in endemic cycles consistent with their places of isolation. Furthermore, homology analysis revealed that nucleotide homology of strains isolated from the southern Asia endemic region was about 94%. However, strains from endemic regions of the eastern Asian coast and central Asia shared more than 96% and 97% homology. Thus, all data above indicate that viruses isolated from southernmost regions of Asia maintained the diversity of virus populations.

**Table 1:** The regional distribution of JEV (genotype 1) isolates in four endemic cycles.

JEV endemic cycles		TMRCA	Isolation time	Southern region					Eastern coastal region				Western region		Central region
				AU	KH	TH	VN	S-MC	E-MC	TW	KR	JP	W-MC	IN	N-MC
Southern endemic cycle	Cluster1	78(52–126)	1969–1979		√	√									
	Cluster2	55(41–77)	1977–2005	√		√	√	√							
Eastern coast endemic cycle	Cluster3	39(33–48)	1979–2003					√	√		√	√			
	Cluster4	33(27–40)	1986–2009						√			√			
	Cluster5	30(25–37)	1991–2010					√	√	√	√	√			√
Western endemic cycle	Cluster6	28(24–35)	1992–2009			√	√					√	√	√	
Central endemic cycle	Cluster7	23(18–31)	1993–2010				√	√	√	√	√	√			√

Note: TMRCA, the most recent common ancestor; KH, Cambodia; TH, Thailand; VN, Vietnam; AU, Australia; S-MC, southern mainland China; E-MC, eastern mainland China; N-MC, northern mainland China; W-MC, western mainland China; TW, Taiwan; KR, South Korea; JP, Japan; IN, India.

In addition, strains isolated from Thailand, Vietnam, and Yunnan Province in China in the same endemic cycle, have a wide span of isolation time. For example, a virus strain isolated in 1979 in Thailand and strains isolated later in 2005 in Thailand were included in the southern Asia endemic cycle. Moreover, the eastern coastal Asia endemic cycle contained strains isolated in 1982 in Yunnan Province in China and others isolated later in 2005 in Yunnan Province; also the western Asia endemic cycle contained strains isolated in Thailand in 1992 and later in 2005; and the central Asia endemic cycle contained strains isolated in Vietnam in 2001 and others isolated in 2007. Thus, in addition to the diversity of virus populations, viruses isolated from the southernmost regions of Asia also maintained the stable genetic characteristics of JEV. This implies that the southernmost region of the Asian continent plays a key role in transmission of JEV from its origin to the Asian continent, providing a source for continental dispersal of JEV strains.

### Dispersal pattern of genotype 1 JEV based on phylogeographic analysis

**Figure 2:**
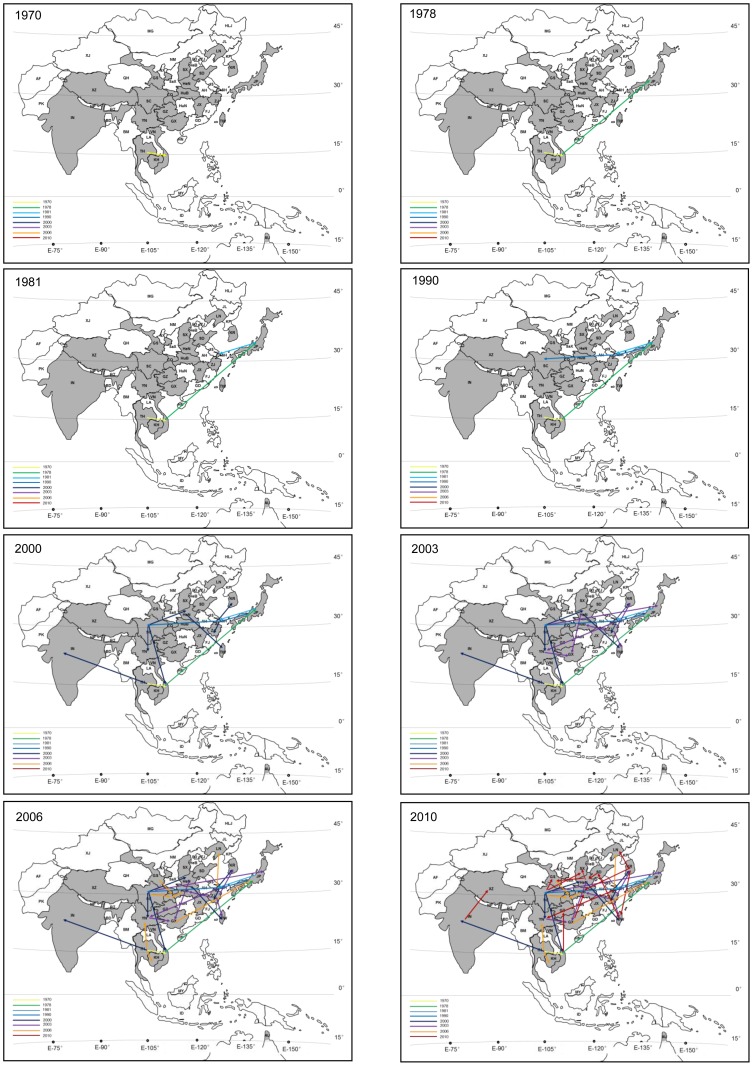
The spatiotemporal migration of Genotype 1 JEV since the 1970s. The different panels represent temporal projections of reconstructed migration events in last 40 years (1970, 1978, 1981, 1990, 2000, 2003, 2006 and 2010, respectively). The different colors, yellow, green, light blue, blue, dark blue, purple, orange, red, dark red demonstrates for 1970, 1978, 1981, 1990, 2000, 2003, 2006 and 2010, respectively. The panels only show the tendency of migration events or partial migration events that have occurred up to a particular date, assuming that the virus migrates at a constant rate over the inferred time span of the branch. Blue circles mark the hot point of migration events. AF, Afghanistan; PK, Pakistan; IN, India; NP, Nepal; BT, Bhutan; BG, Bangladesh; BM, Burma; TH, Thailand; LA, Laos; VN, Vietnam; KH, Cambodia; MY, Malaysia; ID, Indonesia; PP, Papua New Guinea; AU, Australia; KP, North Korea; KR, South Korea; JP, Japan. Chinese provinces: HLJ, Heilongjiang Province; JL, Jilin Province; LN, Liaoning Province; NM, Neimenggu; XJ, Xinjiang; BJ, Beijing, TJ, Tianjin; HeB, Hebei Province; SX, Shanxi Province; SaX, Shaanxi Province; GS, Gansu Province; QH, Qinghai Province; NX, Ningxia; SD, Shandong Province; SH, Shanghai; JS, Jiangsu Province; AH, Anhui Province; HeN, Henan Province; XZ, Xizang; ZJ, Zhejiang Province; JX, Jiangxi Province; HuB, Hubei Province; CQ, Chongqing; SC, Sichuan Province; HuN, Hunan Province; GZ, Guizhou Province; YN, Yunnan Province; FJ, Fujian Province; GD, Guangdong Province; GX, Guangxi; HN, Hainan; TW, Taiwan; MG, Mongolia.

The estimated history of JEV dispersal in endemic regions is shown in detail in Figure 2. The maps in Figure 2 display dispersal characteristics over time. According to our reconstructions, G1 JEV was initially introduced to Thailand and Vietnam located in southernmost Asia during the 1970s after originating in Malaysia/Indonesia (Figure 2, 1970). It then dispersed to Japan and Shanghai, located in east coastal Asia around 1980 (Figure 2, 1978, 1981). Subsequently, the virus was introduced to Sichuan province located in Central China from east coastal Asia around 1990 (Figure 2, 1990). Simultaneously, it spread to India located in the West part of Asia. In 2000, two lineages were dispersed to east coastal Asia (Zhejiang, Liaoning, Taiwan, South Korea) and to the Chinese inland provinces (Henan, Hubei, Gansu and Guizhou), respectively (Figure 2, 2000). After 2000, in addition to continuing its dispersion in east coastal Asia and in Chinese inland provinces, a lineage from Yunnan was introduced to the eastern coastal areas (Figure 2, 2003, 2006). On the other hand, a lineage from Vietnam was introduced to inland China and also from Thailand to Yunnan (Figure 2, 2006). Around 2009, the G1 genotype appears to have dispersed from India to Xizang (Figure 2, 2010). This virus dispersal history supports our contention that the southernmost regions of the Asian continent acted as the source of JEV prior to its transmission throughout the Asian continent.

**Figure 3:**
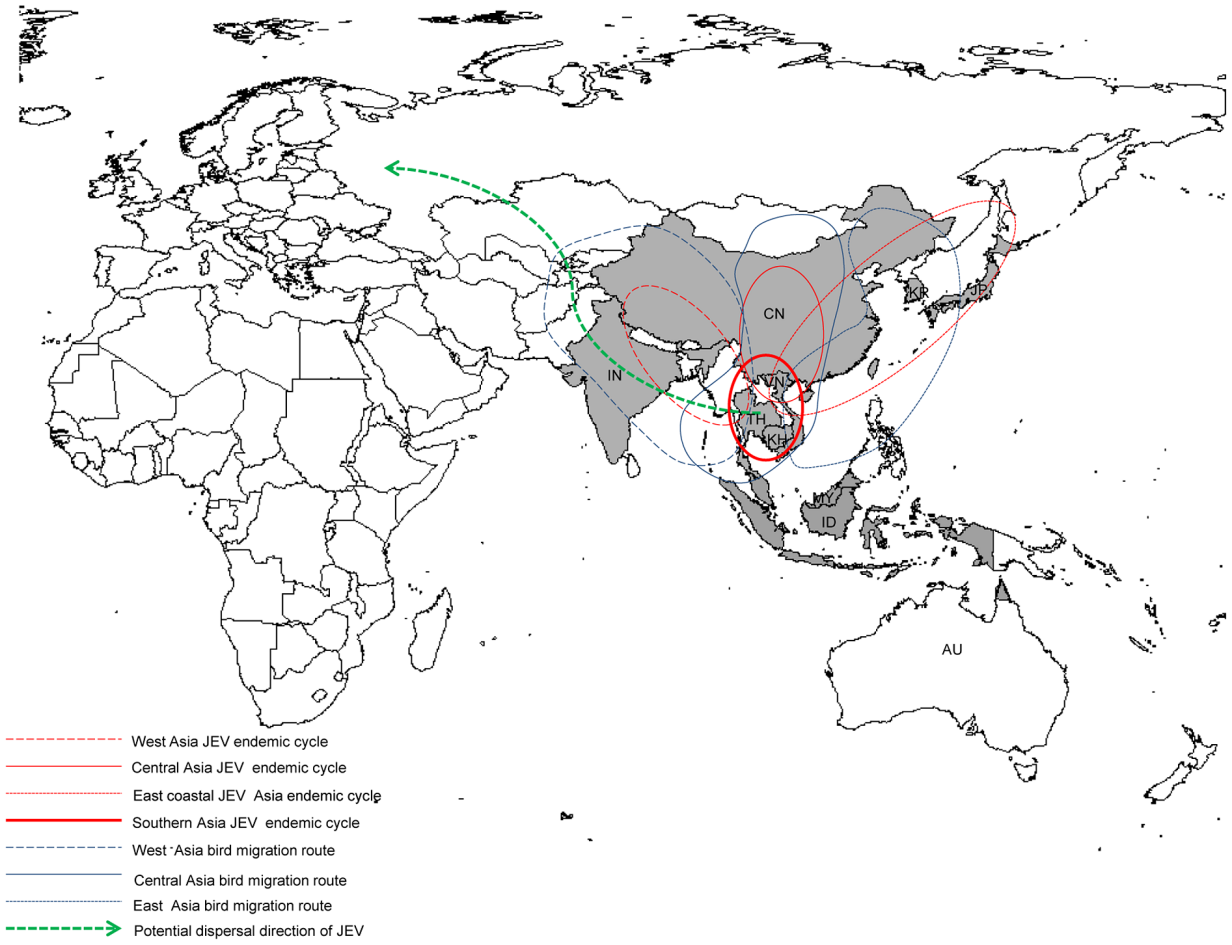
Dispersal characteristics of genotype 1 JEV based on phylogenetic analysis. Gray shadow presents the location of JEV strains used in this study; emboldened circle represents southern Asia endemic cycle established in the JEV transmission source area; red circles presented as dashed lines, intact lines circle and square dot lines represent west Asia, central Asia and east coastal Asia JEV endemic cycles, respectively; blue boxes presented as dashed lines, intact lines and square dots represent bird migration routes in Asia and they coincide with JEV endemic cycles; green dotted lines illustrate the potential dispersal directions of JEV in the future. CN, China; JP, Japan; KR, South Korea; IN, India; VN, Vietnam; TH, Thailand; KH, Cambodia; MY, Malaysia; ID, Indonesia; AU, Australia.

Furthermore, by combining the divergence time and geographical distribution characteristics of each cluster (Table 1) within the MCC tree, three different routes from southern to northern Asia were postulated for dispersal of G1 JEV (Figure 3) Through the eastern route, JEV dispersed from Thailand and Yunnan Province in China, to Japan, South Korea, and Shanghai, Zhejiang, and Liaoning in China generating the eastern coastal Asia endemic cycle. Through the western route, JEV dispersed from Thailand and Vietnam to the western regions of Asia, reaching India and Tibet, establishing the western Asia endemic cycle. Through the central route, JEV was transmitted from southern countries such as Vietnam, to central Asia, and reached inland provinces of China including Sichuan, Chongqing, Guizhou, Hubei, Henan, Gansu, and Shanxi. In addition, JEV was introduced to inland China provinces from bordering east coastal regions and established the largest central Asia endemic cycle.

### Population dynamics of genotype 1 JEV

**Figure 4:**
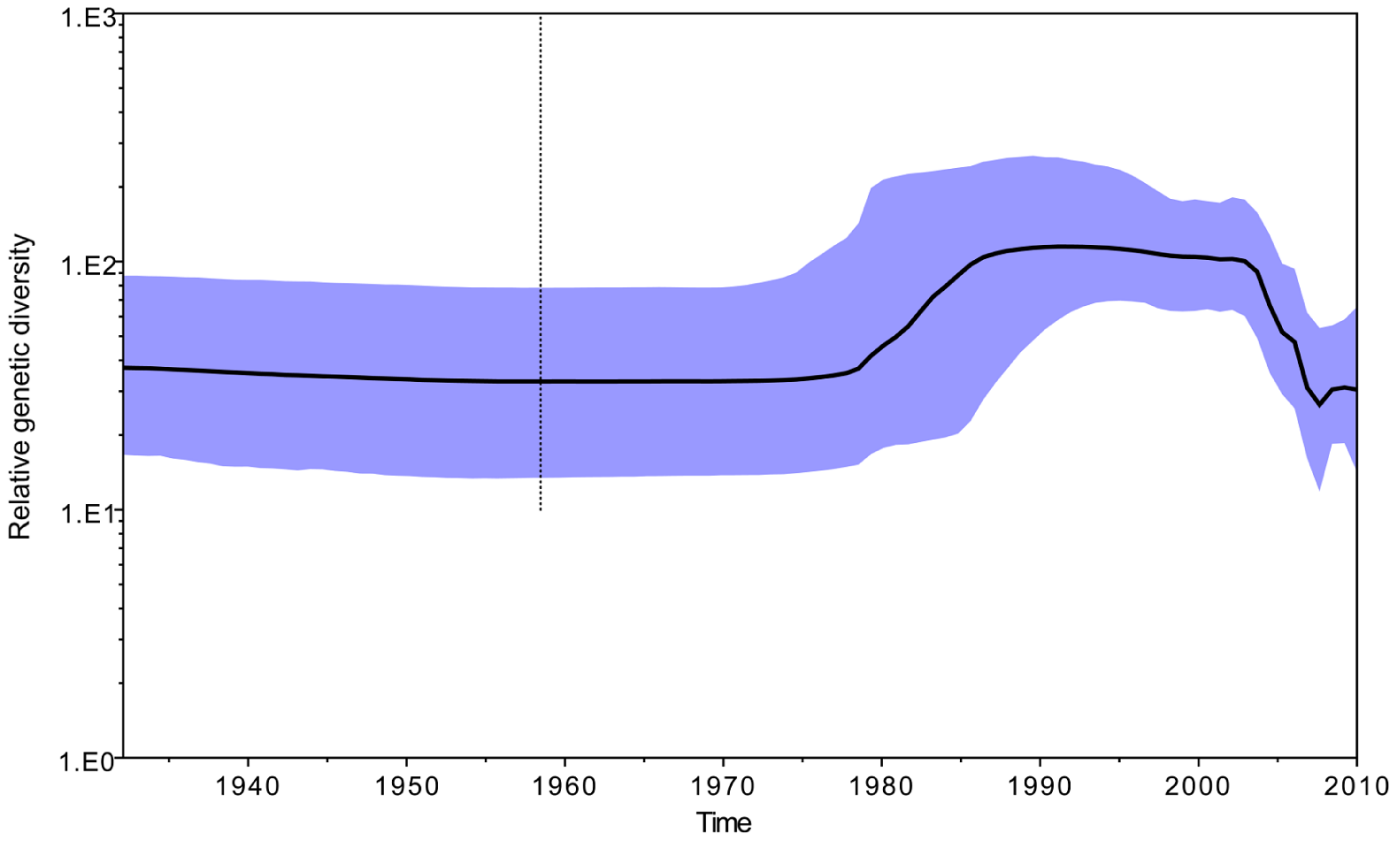
Bayesian skyline plots for genotype 1 JEV. Highlighted areas correspond to 95% HPD intervals.

A skyline plot of the G1 JEV genotype population dynamics is shown in Figure 4. There was minimal fluctuation during the first half of the plot. This was followed by a major population increase from 1980 to 1990, a relatively stable period from 1991 to 2003, a marked decrease during 2004–2007, and then a relatively stable period after 2008.

According to the time nodes of change in population dynamics identified in the skyline plot, the isolation times of JEV in the four endemic cycles were analyzed (Table 2). Viruses were all isolated from the southernmost regions of Asia (Thailand, Cambodia, and Yunnan Province in China) before 1990 during the major period of population increase. Subsequently, from 1991 to 2003, a period of population stability, G1 JEV was found in eastern coastal and southernmost regions of Asia, such as Shanghai and Liaoning in China, Japan, and South Korea. Although there was a virus population decline from 2004 to 2007, the dominant G1 JEV genotype continued its expansion to the central Asian areas. From 2008, the G1 JEV genotype dispersed to all endemic regions in the entire Asian continent and maintained a relatively stable population. JEV continued to be isolated in the southernmost regions of Asia during the entire fluctuation process of virus populations from the first occurrence. In conclusion, all the data are consistent with the concept that the southernmost regions of the Asian continent played a key role as the source for evolution and dispersal of the G1 JEV genotype.

**Table 2:** Temporal distribution of JEV (genotype 1) in four endemic cycles (based on the time spots given by skyline plots).

Region	Country	–1990	1991–2003	2004–2007	2008–2010
Southern region					
	Thailand	√	√	√	
	Vietnam		√	√	
	Cambodia	√			
	Malaysia		√		
	Yunnan	√	√	√	√
	Australia		√		
Eastern coastal region					
	Japan		√	√	√
	South Korea		√	√	√
	Taiwan (China)				√
	Shanghai		√	√	
	Guangxi			√	
	Zhejiang			√	√
	Shandong				√
	Liaoning		√	√	√
Western region					
	India				√
	Xizang				√
Central region					
	Chongqing				√
	Hubei				√
	Gansu			√	√
	Henan			√	
	Guizhou			√	
	Sichuan			√	√
	Jiangxi				√
	Shanxi			√	√

## Discussion

Based on phylogenetic and phylogeographic analysis of the envelope gene of G1 JEV, the following conclusions were drawn from this study. 1) Southernmost Asia, particularly Thailand, Vietnam, and Yunnan Province in China, appear to represent the source for the continental dispersion of JEV which appears to have originated from the Indonesia/Malaysia region; 2) During the dispersal of G1 JEV, limited introductions were observed among difference geographic locations; and 3) Reverse dispersion from north to south occurred during the more recent years. The rationale behind the concept that southernmost Asia might be the source of JEV G1 can be explained as follows. Lying in the tropics and subtropics, these southern regions have a high annual average temperature and heavy annual rainfall both of which are particularly suitable conditions for high population density breeding of a wide range of mosquitoes [24]. *C. tritaeniorhynchus*, the predominant transmission vector for JEV, is widely distributed in Thailand and Vietnam with estimated coverage of 80.9% and 60%, respectively [10]. Additionally, following the remarkable increase in the acreage of irrigated rice in recent years, the distribution of *C. tritaeniorhynchus* has further expanded in Thailand and Vietnam which are traditional rice exporting countries [1]. Furthermore, swine husbandry in these regions has developed rapidly with the number of farmed pigs increasing by 100% from 1990 to 2005 [1]. In conclusion, the combination of a suitable climate year-round, wide distribution of the primary vector and abundant pig farming, provide the ideal prerequisite for the emergence, maintenance and reproduction of JEV in these southernmost regions of Asia.

In addition to these factors, examination of the recognized flight paths of migratory birds reveals a remarkable coincidence between the eastern, central, and western routes of JEV dispersal patterns and the recognized eastern, central, and western flight paths of migratory birds in Asia (Figure 3). In previous studies [11], [12], migratory birds were shown to be important hosts for introducing JEV to new territories. The black-crowned night heron (*Nycticorax nycticorax*), plumed egret (*Egretta intermedia*), and little Egret (*Egretta garzetta*) are the main birds that carry and spread JEV [11], [25]. These observations are consistent with the opinion that migratory birds carrying JEV migrate from south to north annually whereas non-migratory or resting birds are fed upon by mosquitoes in their local habitats. JEV is then further dispersed by local mosquitoes acquiring JEV from the infected birds and transmitting it to domestic pigs which then amplify the virus and provide a local source of infection when local mosquitoes feed on the infected pigs. After a period of evolution and dispersal, dominant JEV populations establish endemic cycles in mosquitoes, pigs and other hosts providing the opportunity for JEV epidemics in local areas. However, taking into account current expert opinion, global climate change could influence the migration patterns or routes of birds, resulting in JEV dispersal into new local areas or even new continents. It is therefore essential to extend JEV surveillance in migrating birds, mosquitoes and pigs in areas currently considered to be free of JEV, including Europe and the New World, where closely related viruses such as West Nile virus (Europe and the Americas) and Usutu virus (Europe) are already known to have established endemic cycles [25], [26]. It was also reported that mosquitoes can be carried very long distances on the wind, especially during the typhoon season [27], [28]. It is therefore conceivable that wind-blown mosquitoes may play a significant role in the dispersal of JEV.

Also, based on studies of the G1 JEV genotype, it is evident that although changes in JEV population dynamics throughout the Asian continent were recorded, a relatively stable state of JEV populations, and wide distribution throughout the Asian continent, was also observed. It therefore seems reasonable to propose that in the absence of adequate control strategies for JEV, the maintenance and widespread distribution of current dominant JEV populations provides a foundation for further expansion of JEV into traditionally non-endemic areas.

In the early and mid-20^th^ century, JEV has caused many pandemics in Asian countries, such as Japan, China and India [2], [29], [30], resulting in huge health, social and economic burdens. With global warming and the increase in acreage of irrigated rice, more Asian regions provide habitats ideal for breeding of *Culex tritaeniorhynchus*, the primary vector of JEV [9]. Additionally, with the changes in pig farming practices and increasing international trade and personnel exchanges, JEV is being provided with numerous opportunities to disperse northwards and westwards to regions outside Asia [31]. For example, a range of *Culex* species is distributed in Europe, such as *Culex pipiens pallens* and *Culex bitaeniorhynchus*. These are recognized transmission vectors of JEV [32]. Moreover, pigs are raised in large areas of north-western European countries [33]. Therefore, if JEV is constantly being introduced to these regions, via infected migratory birds or transportation of infected mosquitoes, during the summer season, the possibility exists for JEV to become established and to form an endemic cycle among the local *Culex* mosquitoes and pigs. Additionally, whilst many viral encephalitic cases in Europe and Asia are known to have been caused by tick-borne encephalitis virus in regions adjacent to Europe [34], JE incidence is currently considered to be extremely low or non-existent in these regions [3]. However, JEV has been detected in specimens collected in the 1990s in the Wooded Steppe region of Northern Eurasia [35] and in avian tissues collected in Russia (GenBank Accession Number AF501313–15). Therefore, it is worth considering the possibility that at least in some cases of encephalitis in Russia and western and central Europe, the aetiological agent may be JEV rather than tick-borne encephalitis. Whilst such a proposal would possibly have been ridiculed a few years ago, several pathogenic mosquito-borne flaviviruses have emerged in Europe during the past decade and recent reports suggest the possibility of the presence of a G3 JEV genotype in birds in Italy [36], [37]. In conclusion, there is clearly a need for a coordinated system of surveillance throughout Europe and Asia in the hope of identifying potential threats of emergence of JEV in new territories.

It is worth noting that, although questions on the transmission pattern of G1 JEV were answered to a certain degree through this study, other questions still require answers. For example, what factors were primarily responsible for the dispersion from Malaysia/Indonesia to Thailand/Yunnan? Why is the JEV G1 genotype no longer detectable in Malaysia/Indonesia? What is the situation regarding the other JEV genotypes? Do they have similar transmission patterns? Further studies will be needed to answer these questions. Our study was based on the available strains. It remains to be seen if, when more data are available, subsequent studies support or revise our conclusions.

## Supporting Information

Table S1 JEV strains used in the present study for phylogenetic analysis.
(DOCX)
