# Supplementary material for: Southernmost Asia Is the Source of Japanese Encephalitis Virus (Genotype 1) Diversity from which the Viruses Disperse and Evolve throughout Asia
Source: PLoS Negl Trop Dis. 2013 Sep 19;7(9):e2459. doi: 10.1371/journal.pntd.0002459 (PMC3777887; doi:10.1371/journal.pntd.0002459)
Supplement: Table S1 — JEV strains used in the present study for phylogenetic analysis. (DOCX) [file pntd.0002459.s001.docx]

**Table S1** JEV strains used in the present study for phylogenetic analysis

| **Strain name** | **Country** | **Region** | **Year** | **Host** | **Genbank** |
| --- | --- | --- | --- | --- | --- |
| M28 | China | Yunnan | 1977 | culex pseudovishinui | JF706279 |
| YN79-Bao83 | China | Yunnan | 1979 | Culex tritaeniorhynchus | DQ404128 |
| YN82-BN8219 | China | Yunnan | 1982 | culex | DQ404129 |
| YN83-Meng83-54 | China | Yunnan | 1983 | Forcipomyia taiwana Shiraki | DQ404130 |
| YN83-83199 | China | Yunnan | 1983 | Culex | DQ404131 |
| YN85-L86-99 | China | Yunnan | 1985 | Culex | DQ404132 |
| YN86-B8639 | China | Yunnan | 1986 | Culex tritaeniorhynchus | DQ404133 |
| YN86-86266 | China | Yunnan | 1986 | na | DQ404134 |
| SH-90 | China | Shanghai | 2001 | Culex tritaeniorhynchus | AY243835 |
| SH-80 | China | Shanghai | 2001 | Culex tritaeniorhynchus | AY243841 |
| SH-53 | China | Shanghai | 2001 | Culex tritaeniorhynchus | AY555757 |
| SH-81 | China | Shanghai | 2001 | Culex tritaeniorhynchus | AY555758 |
| SH-83 | China | Shanghai | 2001 | Culex tritaeniorhynchus | AY555759 |
| SH-96 | China | Shanghai | 2001 | Culex tritaeniorhynchus | AY555760 |
| SH-101 | China | Shanghai | 2001 | Culex tritaeniorhynchus | AY555761 |
| LN02-102 | China | Liaoning | 2002 | Culex modestus | DQ404085 |
| LN02-104 | China | Liaoning | 2002 | Culex pipiens pallens | DQ404086 |
| SH03-103 | China | Shanghai | 2003 | Culex tritaeniorhynchus | DQ404096 |
| SH03-105 | China | Shanghai | 2003 | Culex tritaeniorhynchus | DQ404097 |
| SH03-109 | China | Shanghai | 2003 | Culex tritaeniorhynchus | DQ404098 |
| SH03-115 | China | Shanghai | 2003 | Culex tritaeniorhynchus | DQ404099 |
| SH03-124 | China | Shanghai | 2003 | Culex tritaeniorhynchus | DQ404100 |
| SH03-127 | China | Shanghai | 2003 | Culex tritaeniorhynchus | DQ404101 |
| SH03-128 | China | Shanghai | 2003 | Culex tritaeniorhynchus | DQ404102 |
| SH03-129 | China | Shanghai | 2003 | Culex tritaeniorhynchus | DQ404103 |
| SH03-130 | China | Shanghai | 2003 | Culex tritaeniorhynchus | DQ404104 |
| SH05-24 | China | Shanghai | 2005 | Culex tritaeniorhynchus | DQ404108 |
| HN04-11 | China | Henan | 2004 | Culex | DQ404087 |
| HN04-21 | China | Henan | 2004 | Culex | DQ404088 |
| HN04-40 | China | Henan | 2004 | Culex | DQ404089 |
| SC04-12 | China | Sichuan | 2004 | Culex | DQ404090 |
| SC04-15 | China | Sichuan | 2004 | Culex tritaeniorhynchus | DQ404091 |
| SC04-16 | China | Sichuan | 2004 | Armigeres | DQ404092 |
| SC04-17 | China | Sichuan | 2004 | Culex tritaeniorhynchus | DQ404093 |
| SC04-25 | China | Sichuan | 2004 | Culex | DQ404094 |
| SC04-27 | China | Sichuan | 2004 | Culex | DQ404095 |
| SC09-X77 | China | Sichuan | 2009 | Culex tritaeniorhynchus | JQ411673 |
| SC09-X08 | China | Sichuan | 2009 | Culex tritaeniorhynchus | JQ411671 |
| SC09-A28 | China | Sichuan | 2009 | Culex tritaeniorhynchus | JQ411669 |
| SC07-847 | China | Sichuan | 2007 | Culex tritaeniorhynchus | JQ411667 |
| SC07-210 | China | Sichuan | 2007 | Culex tritaeniorhynchus | JQ411665 |
| SC07-145 | China | Sichuan | 2007 | Culex tritaeniorhynchus | JQ411663 |
| SC07-125 | China | Sichuan | 2007 | Culex tritaeniorhynchus | JQ411662 |
| SC09-X29 | China | Sichuan | 2009 | Culex tritaeniorhynchus | JQ411672 |
| SC09-A38 | China | Sichuan | 2009 | Culex tritaeniorhynchus | JQ411670 |
| SC08-065 | China | Sichuan | 2008 | Culex tritaeniorhynchus | JQ411668 |
| SC07-831 | China | Sichuan | 2007 | Culex tritaeniorhynchus | JQ411666 |
| SC07-146 | China | Sichuan | 2007 | Culex tritaeniorhynchus | JQ411664 |
| SC07-126 | China | Sichuan | 2007 | Culex tritaeniorhynchus | JQ411662 |
| YN05124 | China | Yunnan | 2005 | Culex tritaeniorhynchus | JF706281 |
| YN05155 | China | Yunnan | 2005 | Culex tritaeniorhynchus | JN381852 |
| YN0591 | China | Yunnan | 2005 | Culex tritaeniorhynchus | JQ937348* |
| GX0558 | China | Guangxi | 2005 | Culex tritaeniorhynchus | FJ161969 |
| GX0523 | China | Guangxi | 2005 | Culex tritaeniorhynchus | FJ161968 |
| GX0519 | China | Guangxi | 2005 | Culex tritaeniorhynchus | FJ161967 |
| YN0686 | China | Yunnan | 2006 | Culex tritaeniorhynchus | JQ937351* |
| YN0623 | China | Yunnan | 2006 | Culex tritaeniorhynchus | JN381836 |
| BL06-50 | China | Guangxi | 2006 | Armigeres obturbans | JF706270 |
| BL06-53 | China | Guangxi | 2006 | Armigeres obturbans | JQ937352* |
| BL06-54 | China | Guangxi | 2006 | Armigeres obturbans | JF706271 |
| HN0621 | China | Henan | 2006 | Culex | JN381830 |
| HN0623 | China | Henan | 2006 | Culex | JQ937349* |
| HN0626 | China | Henan | 2006 | Culex | JN381837 |
| HN06129 | China | Henan | 2006 | Armigeres | JF706277 |
| GZ0656 | China | Guizhou | 2006 | CSF | HM366552 |
| LY5 (ly27) | China | Shanxi | 2006 | Mosquito | JQ937345* |
| LN07-02 | China | Liaoning | 2007 | Culex tritaeniorhynchus | JQ937353* |
| LN07-16 | China | Liaoning | 2007 | Culex tritaeniorhynchus | JN381849 |
| GS07-TS11 | China | Gansu | 2007 | Culex tritaeniorhynchus | JN381843 |
| XJ69 | China | Zhejiang | 2007 | Culex pipiens pallens | EU880214 |
| XJP613 | China | Zhejiang | 2007 | Culex tritaeniorhynchus | EU693899 |
| SH17M-2007 | China | Shanghai | 2007 | Culex pipiens pallens | EU429297 |
| ZJ09_52_Zhejiang_09 | China | Zhejiang | 2009 | mosquito | JN216865 |
| ZJ09_108_Zhejiang_09 | China | Zhejiang | 2009 | mosquito | JN216866 |
| ZJ10_23_Zhejiang_10 | China | Zhejiang | 2010 | mosquito | JN216869 |
| ZJ10_45_Zhejiang_10 | China | Zhejiang | 2010 | mosquito | JN216870 |
| ZJ10_7_Zhejiang_10 | China | Zhejiang | 2010 | mosquito | JN216867 |
| ZJ10_10_Zhejiang_10 | China | Zhejiang | 2010 | mosquito | JN216868 |
| 131v | China | Guangxi | 2007 | CSF | GU205163 |
| HEN0701 | China | Henan | 2007 | swine brain | FJ495189 |
| JX61 | China | Zhejiang | 2008 | swine | GU556217 |
| JX66 | China | Zhejiang | 2008 | swine | FJ179364 |
| JX67 | China | Zhejiang | 2008 | swine | FJ179365 |
| GSBY801 | China | Gansu | 2008 | Culex tritaeniorhynchus | JF706274 |
| GSBY804 | China | Gansu | 2008 | Culex tritaeniorhynchus | JN381844 |
| GUBY810 | China | Gansu | 2008 | Culex tritaeniorhynchus | JN381840 |
| GSBY816 | China | Gansu | 2008 | Culex tritaeniorhynchus | JN381842 |
| GSBY827 | China | Gansu | 2008 | Culex tritaeniorhynchus | JN381845 |
| GSBY861 | China | Gansu | 2008 | Culex tritaeniorhynchus | JN381833 |
| LN0828 | China | Liaoning | 2008 | Culex tritaeniorhynchus | JQ937354* |
| HBZG08-09 | China | Hubei | 2008 | Culex tritaeniorhynchus | JQ937333* |
| HBZG-8-59 | China | Hubei | 2008 | Culex tritaeniorhynchus | JQ937340* |
| CQYB08-22 | China | Hubei | 2008 | Culex tritaeniorhynchus | JQ937341* |
| SD08-10 | China | Shandong | 2008 | Culex tritaeniorhynchus | JF706286 |
| DY0821 | China | Shandong | 2008 | Culex tritaeniorhynchus | JQ937338* |
| JN0807 | China | Shangdong | 2008 | Culex tritaeniorhynchus | JQ937339* |
| JN0908 | China | Shandong | 2009 | Culex tritaeniorhynchus | JQ937344* |
| HBZG09-07 | China | Hubei | 2009 | Culex tritaeniorhynchus | JQ937343* |
| HBZG09-36 | China | Hubei | 2009 | Culex tritaeniorhynchus | JQ937336* |
| CQYB09-08 | China | Chongqing | 2009 | Culex tritaeniorhynchus | JQ937337* |
| CQWZ09-13 | China | Chongqing | 2009 | Culex tritaeniorhynchus | JQ937342* |
| JX0939 | China | Jiangxi | 2009 | Culex tritaeniorhynchus | JQ937355* |
| XZ0938 | China | Xizang | 2009 | Mosquito | HQ652538 |
| YN0911 | China | Yunnan | 2009 | Culex tritaeniorhynchus | JF706267 |
| YN0967 | China | Yunnan | 2009 | Culex tritaeniorhynchus | JF706268 |
| LX10P-09 | China | Yunnan | 2009 | CSF | HM204528 |
| LX29P-09 | China | Yunnan | 2009 | CSF | HM204529 |
| LY5P-09 | China | Shanxi | 2009 | CSF | HM204530 |
| JEV-CZ1 | China | Sichuan | 2009 | Culex | HM234673 |
| WHJX9-09 | China | Hubei | 2009 | Culex | HQ437283 |
| WHJX10-09 | China | Hubei | 2009 | Culex | HQ538843 |
| SNJ | China | Hubei | 2010 | Armigeres subalbatus | JQ937346* |
| ES57 | China | Hubei | 2010 | Culex tritaeniorhynchus | JQ937347* |
| JL18 | China | Hubei | 2010 | Culex tritaeniorhynchus | JQ937350* |
| SZ18 | China | Hubei | 2010 | Culex tritaeniorhynchus | JQ937356* |
| Ishikawa | Japan | Ishikawa | 1994 | Culex tritaeniorhynchus | AB051292 |
| JaTAn-1-94 | Japan | Tokyo | 1994 | swine | AB237171 |
| 95-91-Japan-1995-swine | Japan | Oita | 1995 | swine | AY377578 |
| 95-167-Japan-1995-swine | Japan | Oita | 1995 | swine | AY377579 |
| 95P99_Japan_95 | Japan | Oita | 1995 | swine | FJ943471 |
| 97P82_Japan_97 | Japan | Oita | 1997 | swine | FJ943472 |
| JEV-wb-Okinawa-1-1998 | Japan | Okinawa | 1998 | wild boar serum | AB306941 |
| JEV-sw-hiroshima-46-1998 | Japan | Hiroshima | 1998 | swine | AB174837 |
| 99P103_Japan_99 | Japan | Oita | 1999 | swine | FJ943473 |
| 99P104_Japan_99 | Japan | Oita | 1999 | swine | FJ943474 |
| JEV-swine-Hiroshima-38-2000 | Japan | Hiroshima | 2000 | swine | AB174838 |
| JEV-sw-Shizuoka-33-2002 | Japan | Shizuoka | 2002 | swine | AB112703 |
| JEV-sw-Shizuoka-39-2002 | Japan | Shizuoka | 2002 | swine | AB112704 |
| JEV-sw-Chiba-88-2002 | Japan | Chiba | 2002 | swine | AB112705 |
| JEV-sw-Kagawa-24-2002 | Japan | Kagawa | 2002 | swine | AB112706 |
| JEV-sw-Kagawa-27-2002 | Japan | Kagawa | 2002 | swine | AB112707 |
| JEV-sw-Mie-41-2002 | Japan | Mie | 2002 | swine | AB112709 |
| JEV-sw-Hiroshima-25-2002 | Japan | Hiroshima | 2002 | swine | AB231465 |
| JaNAr0102-Japan-2002-Mosquito | Japan | Nagasaki | 2002 | mosquito | AY377577 |
| Oki431S | Japan | Okinawa | 2002 | swine | DQ355369 |
| JEV-eq-Tottori-2003 | Japan | Tottori | 2003 | equine | AB213007 |
| JEV-sw-Okinawa-285-2003 | Japan | Okinawa | 2003 | swine | AB238693 |
| 03P113 | Japan | Oita | 2003 | swine | FJ943475 |
| 03P120 | Japan | Oita | 2003 | swine | FJ943476 |
| 03P126 | Japan | Oita | 2003 | swine | FJ943477 |
| 03P145 | Japan | Oita | 2003 | swine | FJ943478 |
| 03P189 | Japan | Oita | 2003 | swine | FJ943479 |
| Oki568S | Japan | Okinawa | 2003 | swine | DQ355370 |
| Oki589S | Japan | Okinawa | 2003 | swine | DQ355372 |
| JEV-sw-Mie-34-2004 | Japan | Mie | 2004 | swine | AB231462 |
| JEV-sw-Mie-40-2004 | Japan | Mie | 2004 | swine | AB231463 |
| JEV-sw-Kagawa-35-2004 | Japan | Kagawa | 2004 | swine | AB231464 |
| JaNAr07-04 | Japan | Nagasaki | 2004 | mosquito | FJ185144 |
| JaNAr10-04 | Japan | Nagasaki | 2004 | mosquito | FJ185145 |
| JaNAr13-04 | Japan | Nagasaki | 2004 | mosquito | FJ185146 |
| JaNAr31-04 | Japan | Nagasaki | 2004 | mosquito | FJ185150 |
| JaNAr32-04 | Japan | Nagasaki | 2004 | mosquito | FJ185151 |
| JaNAr38-04 | Japan | Nagasaki | 2004 | mosquito | FJ185152 |
| 05231v | Japan | Toyama | 2005 | Sus scrofa domesticus | AB538824 |
| 05197v | Japan | Toyama | 2005 | Sus scrofa domesticus | AB538823 |
| 1256v | Japan | Toyama | 2005 | Culex tritaeniorhynchus | AB538619 |
| 1256c | Japan | Toyama | 2005 | Culex tritaeniorhynchus | AB538618 |
| 1222v | Japan | Toyama | 2005 | Culex tritaeniorhynchus | AB538617 |
| 1222c | Japan | Toyama | 2005 | Culex tritaeniorhynchus | AB538616 |
| 1161v | Japan | Toyama | 2005 | Culex tritaeniorhynchus | AB538615 |
| 1161c | Japan | Toyama | 2005 | Culex tritaeniorhynchus | AB538614 |
| 1160c | Japan | Toyama | 2005 | Culex tritaeniorhynchus | AB538613 |
| 1158v | Japan | Toyama | 2005 | Culex tritaeniorhynchus | AB538612 |
| 1158c | Japan | Toyama | 2005 | Culex tritaeniorhynchus | AB538611 |
| 1157c | Japan | Toyama | 2005 | Culex tritaeniorhynchus | AB538610 |
| 1155v | Japan | Toyama | 2005 | Culex tritaeniorhynchus | AB538609 |
| 1155c | Japan | Toyama | 2005 | Culex tritaeniorhynchus | AB538608 |
| 1149c | Japan | Toyama | 2005 | Culex tritaeniorhynchus | AB538607 |
| 1148v | Japan | Toyama | 2005 | Culex tritaeniorhynchus | AB538606 |
| 1148c | Japan | Toyama | 2005 | Culex tritaeniorhynchus | AB538605 |
| 1089v | Japan | Toyama | 2005 | Culex tritaeniorhynchus | AB538604 |
| 1089c | Japan | Toyama | 2005 | Culex tritaeniorhynchus | AB538603 |
| 1018v | Japan | Toyama | 2005 | Culex tritaeniorhynchus | AB538602 |
| 05P75 | Japan | Oita | 2005 | swine | FJ943480 |
| 1018c | Japan | Toyama | 2005 | Culex tritaeniorhynchus | AB538601 |
| 06P212 | Japan | Oita | 2006 | swine | FJ943484 |
| 06P183 | Japan | Oita | 2006 | swine | FJ943483 |
| 06P169 | Japan | Oita | 2006 | swine | FJ943482 |
| 06P152 | Japan | Oita | 2006 | swine | FJ943481 |
| Sw/Toyama/07326c/2007 | Japan | Toyama | 2007 | swine | AB538831 |
| Sw/Toyama/07296c/2007 | Japan | Toyama | 2007 | swine | AB538830 |
| Sw/Toyama/07292v/2007 | Japan | Toyama | 2007 | swine | AB538829 |
| Sw/Toyama/07292c/2007 | Japan | Toyama | 2007 | swine | AB538828 |
| Sw/Toyama/07240c/2007 | Japan | Toyama | 2007 | swine | AB538827 |
| Sw/Toyama/07234c/2007 | Japan | Toyama | 2007 | swine | AB538826 |
| Sw/Toyama/07232c/2007 | Japan | Toyama | 2007 | swine | AB538825 |
| Mo/Toyama/2569v/2007 | Japan | Toyama | 2007 | Culex tritaeniorhynchus | AB538671 |
| Mo/Toyama/2569c/2007 | Japan | Toyama | 2007 | Culex tritaeniorhynchus | AB538670 |
| Mo/Toyama/2567c/2007 | Japan | Toyama | 2007 | Culex tritaeniorhynchus | AB538669 |
| Mo/Toyama/2556v/2007 | Japan | Toyama | 2007 | Culex tritaeniorhynchus | AB538668 |
| Mo/Toyama/2556c/2007 | Japan | Toyama | 2007 | Culex tritaeniorhynchus | AB538667 |
| Mo/Toyama/2554v/2007 | Japan | Toyama | 2007 | Culex tritaeniorhynchus | AB538666 |
| Mo/Toyama/2554c/2007 | Japan | Toyama | 2007 | Culex tritaeniorhynchus | AB538665 |
| Mo/Toyama/2513v/2007 | Japan | Toyama | 2007 | Culex tritaeniorhynchus | AB538664 |
| Mo/Toyama/2513c/2007 | Japan | Toyama | 2007 | Culex tritaeniorhynchus | AB538663 |
| Mo/Toyama/2507c/2007 | Japan | Toyama | 2007 | Culex tritaeniorhynchus | AB538662 |
| Mo/Toyama/2506c/2007 | Japan | Toyama | 2007 | Culex tritaeniorhynchus | AB538661 |
| Mo/Toyama/2462c/2007 | Japan | Toyama | 2007 | Culex tritaeniorhynchus | AB538660 |
| Mo/Toyama/2441c/2007 | Japan | Toyama | 2007 | Culex tritaeniorhynchus | AB538659 |
| Mo/Toyama/2347c/2007 | Japan | Toyama | 2007 | Culex tritaeniorhynchus | AB538658 |
| JaNAr17-07 | Japan | Nagasaki | 2007 | mosquito | FJ185149 |
| JaNAr15-07 | Japan | Nagasaki | 2007 | mosquito | FJ185148 |
| JaNAr14-07 | Japan | Nagasaki | 2007 | mosquito | FJ185147 |
| JaNAr06-07 | Japan | Nagasaki | 2007 | mosquito | FJ185146 |
| 07P127 | Japan | Oita | 2007 | swine | FJ943487 |
| 07P90 | Japan | Oita | 2007 | swine | FJ943486 |
| 07P83 | Japan | Oita | 2007 | swine | FJ943485 |
| Sw/Toyama/08253c/2008 | Japan | Toyama | 2008 | swine | AB538832 |
| Mo/Toyama/2987v/2008 | Japan | Toyama | 2008 | Culex tritaeniorhynchus | AB538740 |
| Mo/Toyama/2987c/2008 | Japan | Toyama | 2008 | Culex tritaeniorhynchus | AB538739 |
| Mo/Toyama/2986v/2008 | Japan | Toyama | 2008 | Culex tritaeniorhynchus | AB538738 |
| Mo/Toyama/2986c/2008 | Japan | Toyama | 2008 | Culex tritaeniorhynchus | AB538737 |
| Mo/Toyama/2985v/2008 | Japan | Toyama | 2008 | Culex tritaeniorhynchus | AB538736 |
| Mo/Toyama/2985c/2008 | Japan | Toyama | 2008 | Culex tritaeniorhynchus | AB538735 |
| Mo/Toyama/2984v/2008 | Japan | Toyama | 2008 | Culex tritaeniorhynchus | AB538734 |
| Mo/Toyama/2984c/2008 | Japan | Toyama | 2008 | Culex tritaeniorhynchus | AB538733 |
| Mo/Toyama/2977v/2008 | Japan | Toyama | 2008 | Culex tritaeniorhynchus | AB538732 |
| Mo/Toyama/2977c/2008 | Japan | Toyama | 2008 | Culex tritaeniorhynchus | AB538731 |
| Mo/Toyama/2976v/2008 | Japan | Toyama | 2008 | Culex tritaeniorhynchus | AB538730 |
| Mo/Toyama/2976c/2008 | Japan | Toyama | 2008 | Culex tritaeniorhynchus | AB538729 |
| Mo/Toyama/2967v/2008 | Japan | Toyama | 2008 | Culex tritaeniorhynchus | AB538728 |
| Mo/Toyama/2967c/2008 | Japan | Toyama | 2008 | Culex tritaeniorhynchus | AB538727 |
| Mo/Toyama/2929v/2008 | Japan | Toyama | 2008 | Culex tritaeniorhynchus | AB538726 |
| Mo/Toyama/2929c/2008 | Japan | Toyama | 2008 | Culex tritaeniorhynchus | AB538725 |
| Mo/Toyama/2918c/2008 | Japan | Toyama | 2008 | Culex tritaeniorhynchus | AB538724 |
| Mo/Toyama/2917c/2008 | Japan | Toyama | 2008 | Culex tritaeniorhynchus | AB538723 |
| Mo/Toyama/2915c/2008 | Japan | Toyama | 2008 | Culex tritaeniorhynchus | AB538722 |
| Mo/Toyama/2910c/2008 | Japan | Toyama | 2008 | Culex tritaeniorhynchus | AB538721 |
| Mo/Toyama/2909c/2008 | Japan | Toyama | 2008 | Culex tritaeniorhynchus | AB538720 |
| Mo/Toyama/2906c/2008 | Japan | Toyama | 2008 | Culex tritaeniorhynchus | AB538719 |
| Mo/Toyama/2905c/2008 | Japan | Toyama | 2008 | Culex tritaeniorhynchus | AB538718 |
| Mo/Toyama/2895c/2008 | Japan | Toyama | 2008 | Culex tritaeniorhynchus | AB538717 |
| Mo/Toyama/2888c/2008 | Japan | Toyama | 2008 | Culex tritaeniorhynchus | AB538716 |
| Mo/Toyama/2886v/2008 | Japan | Toyama | 2008 | Culex tritaeniorhynchus | AB538715 |
| Mo/Toyama/2886c/2008 | Japan | Toyama | 2008 | Culex tritaeniorhynchus | AB538714 |
| Mo/Toyama/2853v/2008 | Japan | Toyama | 2008 | Culex tritaeniorhynchus | AB538713 |
| Mo/Toyama/2853c/2008 | Japan | Toyama | 2008 | Culex tritaeniorhynchus | AB538712 |
| Mo/Toyama/2842v/2008 | Japan | Toyama | 2008 | Culex tritaeniorhynchus | AB538711 |
| Mo/Toyama/2842c/2008 | Japan | Toyama | 2008 | Culex tritaeniorhynchus | AB538710 |
| Mo/Toyama/2821c/2008 | Japan | Toyama | 2008 | Culex tritaeniorhynchus | AB538709 |
| Mo/Toyama/2808c/2008 | Japan | Toyama | 2008 | Culex tritaeniorhynchus | AB538708 |
| Mo/Toyama/2805v/2008 | Japan | Toyama | 2008 | Culex tritaeniorhynchus | AB538707 |
| Mo/Toyama/2805c/2008 | Japan | Toyama | 2008 | Culex tritaeniorhynchus | AB538706 |
| Mo/Toyama/2795v/2008 | Japan | Toyama | 2008 | Culex tritaeniorhynchus | AB538705 |
| Mo/Toyama/2795c/2008 | Japan | Toyama | 2008 | Culex tritaeniorhynchus | AB538704 |
| Mo/Toyama/2794v/2008 | Japan | Toyama | 2008 | Culex tritaeniorhynchus | AB538703 |
| Mo/Toyama/2794c/2008 | Japan | Toyama | 2008 | Culex tritaeniorhynchus | AB538702 |
| Mo/Toyama/2759c/2008 | Japan | Toyama | 2008 | Culex tritaeniorhynchus | AB538701 |
| Mo/Toyama/2757c/2008 | Japan | Toyama | 2008 | Culex tritaeniorhynchus | AB538700 |
| TPC0806c | Taiwan | Taipei city | 2008 | Culex tritaeniorhynchus | GQ260635 |
| YL0806f | Taiwan | Yulin county | 2008 | Culex tritaeniorhynchus | GQ260633 |
| TC2009-4 | Taiwan | Taichuang county | 2009 | mosquito | JF499794 |
| YL2009-5 | Taiwan | Yulin county | 2009 | mosquito | JF499809 |
| YL2009-3 | Taiwan | Yulin county | 2009 | mosquito | JF499807 |
| YL2009-2 | Taiwan | Yulin county | 2009 | mosquito | JF499806 |
| YL2009-4 | Taiwan | Yulin county | 2009 | mosquito | JF499808 |
| TC2009-10 | Taiwan | Taichuang county | 2009 | mosquito | JF499800 |
| TC2009-13 | Taiwan | Taichuang county | 2009 | mosquito | JF499803 |
| YL2009-1 | Taiwan | Yulin county | 2009 | mosquito | JF499805 |
| TC2009-3 | Taiwan | Taichuang county | 2009 | mosquito | JF499793 |
| TC2009-14 | Taiwan | Taichuang county | 2009 | mosquito | JF499804 |
| TC2009-12 | Taiwan | Taichuang county | 2009 | mosquito | JF499802 |
| TC2009-7 | Taiwan | Taichuang county | 2009 | mosquito | JF499797 |
| TC2009-1 | Taiwan | Taichuang county | 2009 | mosquito | JF499791 |
| TC2009-9 | Taiwan | Taichuang county | 2009 | mosquito | JF499799 |
| TC2009-5 | Taiwan | Taichuang county | 2009 | mosquito | JF499795 |
| TC2009-8 | Taiwan | Taichuang county | 2009 | mosquito | JF499798 |
| TC2009-2 | Taiwan | Taichuang county | 2009 | mosquito | JF499792 |
| TC2009-6 | Taiwan | Taichuang county | 2009 | mosquito | JF499796 |
| TC2009-11 | Taiwan | Taichuang county | 2009 | mosquito | JF499801 |
| CY2010-3 | Taiwan | Chiayi county | 2010 | mosquito | JF499824 |
| CY2010-2 | Taiwan | Chiayi county | 2010 | mosquito | JF499823 |
| CY2010-1 | Taiwan | Chiayi county | 2010 | mosquito | JF499822 |
| YL2010-1 | Taiwan | Yulin county | 2010 | mosquito | JF499816 |
| YL2010-2 | Taiwan | Yulin county | 2010 | mosquito | JF499817 |
| CY2010-4 | Taiwan | Chiayi county | 2010 | mosquito | JF499825 |
| TC2010-5 | Taiwan | Taichuang county | 2010 | mosquito | JF499814 |
| TC2010-2 | Taiwan | Taichuang county | 2010 | mosquito | JF499811 |
| TC2010-6 | Taiwan | Taichuang county | 2010 | mosquito | JF499815 |
| YL2010-3 | Taiwan | Yulin county | 2010 | mosquito | JF499818 |
| CH2010-3 | Taiwan | Changhua county | 2010 | mosquito | JF499821 |
| HL2010-1 | Taiwan | Hualien county | 2010 | mosquito | JF499827 |
| TN2010-1 | Taiwan | Tainan county | 2010 | mosquito | JF499826 |
| CH2010-2 | Taiwan | Changhua county | 2010 | mosquito | JF499820 |
| CH2010-1 | Taiwan | Changhua county | 2010 | mosquito | JF499819 |
| TC2010-3 | Taiwan | Taichuang county | 2010 | mosquito | JF499812 |
| TC2010-1 | Taiwan | Taichuang county | 2010 | mosquito | JF499810 |
| TC2010-4 | Taiwan | Taichuang county | 2010 | mosquito | JF499813 |
| 08P62 | Japan | Oita | 2008 | swine | FJ943494 |
| 08P54 | Japan | Oita | 2008 | swine | FJ943493 |
| 08P49 | Japan | Oita | 2008 | swine | FJ943492 |
| 08P48 | Japan | Oita | 2008 | swine | FJ943491 |
| 08P42 | Japan | Oita | 2008 | swine | FJ943490 |
| 08P38 | Japan | Oita | 2008 | swine | FJ943489 |
| 08P37 | Japan | Oita | 2008 | swine | FJ943488 |
| noname | Japan | Nishinomiya | 2008 | Sus scrofa leucomystax | AB481224 |
| JEV/sw/Okinawa/402/2008 | Japan | Okinawa | 2008 | swine | AB471670 |
| JEV/sw/Okinawa/377/200 | Japan | Okinawa | 2008 | swine | AB471669 |
| JEV/sw/Okinawa/372/2008 | Japan | Okinawa | 2008 | swine | AB471668 |
| JEV/sw/Okinawa/254/2008 | Japan | Okinawa | 2008 | swine | AB471667 |
| JEV/sw/Okinawa/154/2008 | Japan | Okinawa | 2008 | swine | AB471666 |
| Mo/Toyama/3141c/2009 | Japan | Toyama | 2009 | Culex tritaeniorhynchus | AB543740 |
| Mo/Toyama/3140c/2009 | Japan | Toyama | 2009 | Culex tritaeniorhynchus | AB543739 |
| Mo/Toyama/3133c/2009 | Japan | Toyama | 2009 | Culex tritaeniorhynchus | AB543738 |
| 09P141 | Japan | Oita | 2009 | swine | GU108335 |
| 09P123 | Japan | Oita | 2009 | swine | GU108334 |
| K91p55 | Korea | Wando | 1991 | Culex tritaeniorhynchus | U34928 |
| K93A07 | Korea | na | 1993 | mosquito | FJ938230 |
| K94A07_Korea_94 | Korea | na | 1994 | mosquito | FJ938216 |
| K94P05 | Korea | Wando | 1994 | Culex tritaeniorhynchus | JEU34929 |
| K95A07_Korea_95 | Korea | na | 1995 | mosquito | FJ938218 |
| K96A07_Korea_96 | Korea | na | 1996 | mosquito | FJ938219 |
| KV1899 | Korea | Gyeonggi | 1999 | swine | AY316157 |
| K01_JB_Korea_2001 | Korea | Jeon-Buk | 2001 | Culex tritaeniorhynchus | FJ938221 |
| K01_JN_Korea_2001 | Korea | Jeon-Nam | 2001 | Culex tritaeniorhynchus | FJ938222 |
| K01_GN_Korea_2001 | Korea | Gyeong-Nam | 2001 | Culex tritaeniorhynchus | FJ938220 |
| K05_GS | Korea | Gunsan | 2005 | Culex tritaeniorhynchus | FJ938223 |
| A8_789 | Korea | Jeonnam | 2008 | Culex tritaeniorhynchus | JN587261 |
| A10_881 | Korea | Gyeongnam | 2010 | Culex tritaeniorhynchus | JN587260 |
| A10_825 | Korea | Gyeongnam | 2010 | Culex tritaeniorhynchus | JN587259 |
| VN88-Viet-Nam-2001-swine | Vietnam | HaTay | 2001 | swine | AY376464 |
| VN22-Viet-Nam-2002-swine | Vietnam | Northern vietnam | 2002 | swine | AY376465 |
| VN34-Viet-Nam-2002-Mosquito | Vietnam | Northern vietnam | 2002 | mosquito | AY376466 |
| VN78-Viet-Nam-2002-Mosquito | Vietnam | HaTay | 2002 | mosquito | AY376467 |
| VN105-Viet-Nam-2002-Mosquito | Vietnam | HaNam | 2002 | mosquito | AY376468 |
| CT_MO_P7 | Vietnam | na | 2005 | swine | HQ009266 |
| LA_H_5330 | Vietnam | na | 2005 | swine | HQ009265 |
| LA-H06-05 | Vietnam | na | 2005 | mosquito | FJ185153 |
| LA-H07-05 | Vietnam | na | 2005 | mosquito | FJ185154 |
| LAH-2079-05 | Vietnam | na | 2005 | mosquito | FJ185155 |
| 07VN311 | Vietnam | Tay Nguyen | 2007 | Culex tritaeniorhynchus | HM228923 |
| 07VN310 | Vietnam | Tay Nguyen | 2007 | Culex tritaeniorhynchus | HM228922 |
| LAM-2.3 | Vietnam | Long an | 2005 | Swine | 窗体顶端  JN574432窗体底端 |
| LAH-5334 | Vietnam | Long an | 2005 | Swine | 窗体顶端  JN574430窗体底端 |
| LAM-01 | Vietnam | Long an | 2005 | Swine | JN574431 |
| LAH-08 | Vietnam | Long an | 2005 | Swine | JN574429 |
| 2372-Thailand-1979-Human | Thailand | na | 1979 | Human | JEU70401 |
| P19Br | Thailand | Chiang Mai | 1982 | Human | U70416 |
| B2239-Thailand-1984-Pig | Thailand | Chiang Mai | 1984 | swine | JEU70391 |
| ThCMAr4492 | Thailand | Chiang Mai | 1992 | Culex tritaeniorhynchus | D45362 |
| ThCMAr6793 | Thailand | na | 1993 | Cx. vishnui | D45363 |
| JE-RT-36 | Thailand | Ratchaburi | 2003 | mosquito | DQ087975 |
| JE-PK52 | Thailand | Phuket | 2004 | mosquito | DQ084229 |
| JE-CP-67 | Thailand | Chumphon | 2004 | swine | DQ087972 |
| JE-CP-49 | Thailand | Chumphon | 2004 | swine | DQ087974 |
| JE-KK-80 | Thailand | KhonKhen | 2004 | swine | DQ111784 |
| JE-KK-82 | Thailand | KhonKhen | 2004 | swine | DQ111785 |
| JE-KK-R88 | Thailand | KhonKhen | 2004 | na | DQ111786 |
| JE-KK-R83 | Thailand | KhonKhen | 2004 | na | DQ111787 |
| JE-KK-R87 | Thailand | KhonKhen | 2004 | na | DQ111788 |
| JE-KK-580 | Thailand | KhonKhen | 2005 | swine | DQ238600 |
| JE-KK-577 | Thailand | KhonKhen | 2005 | swine | DQ238601 |
| JE-CM-1196 | Thailand | ChangMai | 2005 | swine | DQ238602 |
| JE-KK-1116 | Thailand | KhonKhen | 2005 | swine | DQ343290 |
| M859-Cambodia-1967-Mosquito | Cambodia | na | 1967 | mosquito | JEU70410 |
| TS00 | Austraria | Torres Strait | 2000 | swine | EF434785 |
| JEV-GKP/0945054 | India | Gorakhpurm | 2009 | CSF | HM156572 |
| JEV-GKP/0951220 | India | Gorakhpurm | 2009 | CSF | HM156571 |
| JEV-GKP/0944400 | India | Gorakhpurm | 2009 | CSF | HM156570 |

Note: na, information not available; asterisks indicate strains newly sequenced in this study.
